# Allogeneic Adipose-Derived Mesenchymal Stromal Cells Ameliorate Experimental Autoimmune Encephalomyelitis by Regulating Self-Reactive T Cell Responses and Dendritic Cell Function

**DOI:** 10.1155/2017/2389753

**Published:** 2017-01-30

**Authors:** Per Anderson, Elena Gonzalez-Rey, Francisco O'Valle, Francisco Martin, F. Javier Oliver, Mario Delgado

**Affiliations:** ^1^Institute of Parasitology and Biomedicine “López-Neyra”, CSIC, PTS, Granada, Spain; ^2^GENYO, Centre of Genomics and Oncological Research, Pfizer/University of Granada/Andalusian Regional Government, PTS, Granada, Spain; ^3^School of Medicine, Department of Pathology, IBIMER, CIBM, University of Granada, Granada, Spain

## Abstract

Multipotent mesenchymal stromal cells (MSCs) have emerged as a promising therapy for autoimmune diseases, including multiple sclerosis (MS). Administration of MSCs to MS patients has proven safe with signs of immunomodulation but their therapeutic efficacy remains low. The aim of the current study has been to further characterize the immunomodulatory mechanisms of adipose tissue-derived MSCs (ASCs) in vitro and in vivo using the EAE model of chronic brain inflammation in mice. We found that murine ASCs (mASCs) suppress T cell proliferation in vitro via inducible nitric oxide synthase (iNOS) and cyclooxygenase- (COX-) 1/2 activities. mASCs also prevented the lipopolysaccharide- (LPS-) induced maturation of dendritic cells (DCs) in vitro. The addition of the COX-1/2 inhibitor indomethacin, but not the iNOS inhibitor L-NAME, reversed the block in DC maturation implicating prostaglandin (PG) E_2_ in this process. In vivo, early administration of murine and human ASCs (hASCs) ameliorated myelin oligodendrocyte protein- (MOG_35-55_-) induced EAE in C57Bl/6 mice. Mechanistic studies showed that mASCs suppressed the function of autoantigen-specific T cells and also decreased the frequency of activated (CD11c^+^CD40^high^ and CD11c^+^TNF-*α*^+^) DCs in draining lymph nodes (DLNs). In summary, these data suggest that mASCs reduce EAE severity, in part, through the impairment of DC and T cell function.

## 1. Introduction

Multipotent mesenchymal stromal cells (MSCs) are nonhematopoietic, perivascular cells which support hematopoiesis and are thought to participate in tissue repair in vivo [1–3]. MSCs can be obtained from virtually all tissues and organs of the body and have been defined in vitro as plastic adherent cells that express CD73, CD90, and CD105 while lacking expression of CD45, CD34, and CD14, which can differentiate into osteoblasts, adipocytes, and chondroblasts in vitro [4]. Although the proportion of* bona fide *stem cells within MSC preparations is likely to be low [5], it has been clearly shown that in vitro-expanded MSCs possess potent immunomodulatory properties. Firstly, MSCs can modulate the activation of cells of both the innate and adaptive immune system in vitro. Depending on species and immune cell studied, this immunomodulatory effect is achieved through a combination of mechanisms including cytokines (IL-10, TGF-*β*1, and IL-6), intracellular enzymes (inducible nitric oxide synthase (iNOS) in murine MSCs and indoleamine 2,3-dioxygenase (IDO) in human MSCs), growth factors (hepatocyte growth factor, vascular endothelial growth factor), membrane bound molecules (programmed death ligand-1, FASL), and prostaglandin E2 (PGE_2_) [6–8]. Secondly, injection of MSC preparations has shown delay allograft rejection [9, 10] and ameliorating disease in several animal models of inflammation/autoimmunity by inhibiting the deleterious immune responses against self-antigens [11–14]. Based on their success in experimental models, the clinical potential of human autologous and allogeneic MSCs for the treatment of autoimmune/inflammatory diseases has been assessed in various phase I/II and III clinical trials [15–19].

Multiple sclerosis (MS) is a chronic disease of the central nervous system (CNS) where autoreactive T cells and macrophages attack and disrupt the communication between neurons which result in a multitude of neurological symptoms [20]. Several clinical trials have utilized MSCs for the treatment of MS and a few of these studies have analyzed the effects of injected MSCs on the immune status of patients with MS. In a phase 1/2 open-safety clinical trial for MS, injections of autologous MSCs resulted in a rapid inhibition of the immune system [17] while another study observed increased foxp3 transcript levels in peripheral blood mononuclear cells in MSC-treated patients [21]. These data suggest that the therapeutic effect of MSCs in MS depends, at least partially, upon their immunomodulatory capacity. However, while the administration of MSCs has been proven safe, their capacity to ameliorate MS remains poor [16, 17, 19, 22]. Therefore, it is important to further study how MSCs can modulate the immune system both in vitro and in vivo in order to improve MSC-based therapies for MS [23].

Thus, the objective of the current study has been to characterize the immunomodulatory properties of murine adipose tissue-derived MSCs (mASC) in vitro and in vivo using the experimental autoimmune encephalomyelitis (EAE) model of CNS autoimmunity. EAE is a well-accepted animal model of MS sharing both its immunological and pathological features [24]. Injection of MSCs at distinct stages of disease has been shown to inhibit disease severity in various models of EAE, including relapsing/remitting EAE, chronic EAE, and disease induced by adoptive transfer of encephalitogenic T cells [11, 25–27]. We show that mASC inhibited T cell proliferation in vitro via iNOS and COX-1/2 activities. Both allogeneic and xenogeneic (human) ASCs ameliorated MOG_35-55_-induced chronic EAE in C57Bl/6 mice. Murine ASCs reduced EAE severity through the inhibition of the autoimmune T cell response with no increase in foxp3 Tregs. Importantly, mASC inhibited the maturation of dendritic cells (DCs) in vitro via COX-1/2 activity and reduced the percentage of activated DCs in the draining LNs of EAE mice. Our data suggests that MSC, through their modulation of T cell and DC function, can ameliorate inflammatory/autoimmune CNS disease.

## 2. Materials and Methods

### 2.1. Animals

Male Balb/c (6–10 weeks) and female C57Bl/6 (6–8 weeks) (Charles River, Barcelona, Spain) were used to initiate cultures of mASCs and for the induction of EAE, respectively. Experimental protocols were performed according to the National/EU Guidelines for the Care and Use of Laboratory Animals in Research with the approval of the local ethical committee at the Institute for Parasitology and Biomedicine “López-Neyra” and the central ethical committee at the Consejo Superior de Investigaciones Cientificas (CSIC). Mice were anesthetized before immunization using intraperitoneal injections of ketamine-HCl (dose at 100 mg/kg) mixed with xylazine-HCl (10 mg/kg). Mice were sacrificed using CO_2_ when extracting the spinal cord for histological analyses. In all other cases, mice were sacrificed using cervical dislocation.

### 2.2. Isolation and Expansion of mASCs

Mesenchymal stromal cells were isolated from adipose tissue as previously described [28]. Cells were resuspended in MesenCult (Stem Cell, Grenoble, France) containing 20% mouse mesenchymal supplements (Stem Cell) and penicillin/streptomycin (Invitrogen, Carlsbad, CA), plated at a density of 2-3 × 10^4^ cells/cm^2^ and cultured at 37°C in hypoxia at 5% O_2_, 5% CO_2_. Nonadherent cells were removed after 24 hours in culture. Subsequent passages were plated at 10^4^ cells/cm^2^ and maintained at 5% O_2_, 5% CO_2_. mASCs were used at passages 3–9. The phenotypic characterization and differentiation capacity into adipocytes, osteocytes, and chondrocytes were performed as previously described ([28]; data not shown). Human ASCs were obtained from Cellerix SA, Tres Cantos (Madrid), Spain, and cultured in advanced DMEM supplemented with 10% FCS (Invitrogen), Glutamax (GIBCO, Life Technologies, CA), and 100 U/mL penicillin/streptomycin (GIBCO, Life Technologies) as previously described [14].

### 2.3. Assessment of mASC-Mediated Inhibition of T Cell Proliferation In Vitro

mASCs were treated with mitomycin C (50 *μ*g/mL, Sigma Aldrich, St. Louis, MO) for 20 minutes at 37°C and washed 3 times with complete RPMI1640 (2 mM L-glutamine, 100 U/mL penicillin/streptomycin, 50 *μ*M 2-mercaptoethanol, and 10% heat-inactivated FCS, all from Invitrogen). Mitomycin C-treated mASCs were plated in flat bottomed 96-well plates and allowed to adhere for 3-4 hours. Mitomycin C treatment of mASCs did not impact on their ability to inhibit T cell proliferation in vitro (see Figure S1 in Supplementary Material available online at https://doi.org/10.1155/2017/2389753). Spleens from female Balb/c mice were homogenized and erythrocytes lysed using ACK buffer (0.15 M NH_4_Cl, 10 mM KHCO_3_, and 0.1 mM Na_2_EDTA at pH 7.4) and 2 × 10^5^ cells were added to wells with or without mASCs. Concanavalin A (ConA, 2.5 *μ*g/mL, Sigma Aldrich) was added to cultures as a mitogenic stimulus of T cells. L-NAME (1 mM), indomethacin (20 *μ*M), and L-norvaline (10 mM) (all from Sigma Aldrich) were added at the initiation of the cocultures. After 3 days, cells were pulsed with 0.5 *μ*Ci/well [^3^H]-thymidine (Perkin Elmer, Waltham, MA) for 6 hours and harvested onto glass fiber filters using a FilterMate 96 well-harvester (Perkin Elmer). Uptake of [^3^H]-thymidine was measured on a 1450 Microbeta Trilux scintillation counter (Wallac Oy, Turku, Finland).

### 2.4. Measurement of Nitrite Production

To assess iNOS activity in mASCs, supernatants from control/stimulated mASCs and mASC: DC cocultures were assayed for nitrite contents using the Griess assay. In brief, 100 *μ*L of Griess reagent (a 1/1 mixture of 1% p-aminobenzene-sulfonamide in 5% H_3_PO_4_ and 0.1% naphthyl ethylenediamine dihydrochloride in distilled H_2_O; Sigma Aldrich) was added to 100 *μ*L culture supernatants and standard (NaNO_2_) in 96-well plates. Plates were incubated at room temperature for 10 min, and absorbance was measured at 550 nm.

### 2.5. Measurement of Arginase Activity

Arginase activity was measured as previously described [29]. Urea was used for the standard curve. The protein concentrations of cell lysates were measured using the bicinchoninic acid (BCA) assay [30]. One unit of enzyme activity is defined as the amount of enzyme that catalyzes the formation of 1 mmol of urea per min.

### 2.6. Induction of EAE

Female C57Bl/6 mice (6–8 weeks-old) were immunized s.c. in the flanks with 150 *μ*L of an emulsion (75 *μ*L/flank) containing 150 *μ*g MOG_35-55_ (Genscript, Hong Kong, China) in PBS mixed with an equal volume of complete Freund's adjuvant (CFA) supplemented with 4 mg/mL* Mycobacterium tuberculosis* H37Ra (Difco, Detroit, MI). Mice were injected i.p. with 200 ng pertussis toxin (Sigma Aldrich) in PBS on the day of immunization and 2 days later. Immunized mice were randomly distributed in different groups. Group 1: control mice (*n* = 8) were injected i.p. with PBS at the onset of disease (clinical scores: 0-1). Group 2: control mice (*n* = 13) were injected i.p. with PBS at the acute phase of disease (clinical scores: 1–3). Group 3: Mice (*n* = 9) were treated i.p. with allogeneic mASCs (10^6^ cells obtained from Balb/c mice and expanded in hypoxia) at the onset (clinical scores: 0-1). Group 4: mice (*n* = 7) were treated i.p. with allogeneic mASCs (10^6^ cells obtained from Balb/c mice and expanded in hypoxia) at the acute phase of the disease (clinical scores: 2-3). Group 5: mice (*n* = 7) were treated i.p. with hASCs (10^6^ cells) at the acute phase of disease (clinical scores: 1-2). Clinical symptoms of EAE were scored daily using a 0–8 scale as follows: 0, no detectable signs of EAE; 1, affected tail tonus; 2, tail paralysis; 3, mild hind leg paresis; 4, severe hind leg paresis; 5, one hind leg paralysis; 6, complete hind leg paralysis; 7, complete hind leg paralysis and foreleg paresis; and 8, death. For the acquisition of cells and tissues, another set of mice were used and sacrificed 7 days after treatment with PBS or mASCs as described below. Mice were scored daily for disease symptoms. Water gel products providing water and moistened food pellets were placed on the cage floor in Petri dishes which were changed daily to prevent dehydration. Mice were euthanized if exhibiting severe hind leg paralysis and foreleg paresis (a clinical score of 7).

### 2.7. Histological Analysis of Cell Infiltration and Demyelinization

Spinal cords from EAE mice treated i.p. with PBS (*n* = 4) or allogeneic mASC (*n* = 4) at the onset of disease (clinical scores: 0-1) were removed 7 days after treatment and processed for immunohistochemistry and Klüver-Barrera staining. For light microscopy, cervical and lumbar spinal cord segments were fixed with buffered 10% formalin for 48 h and processed for paraffin inclusion and sectioning. Transversal sections (4 *μ*m thickness) were stained with Luxol fast blue, cresyl violet, and hematoxylin following the Klüver-Barrera technique [31] and were analyzed for the presence of areas of demyelination and cell infiltration using a light microscope (Olympus, Tokyo, Japan). For immunohistochemistry, spinal cord sections were obtained as described for paraffin processing followed by blocking steps with peroxidase blocking reagents, heat-treated in 1 mM EDTA buffer pH 8 at 95°C during 20 min for antigenic unmasking, and incubated for 30 min at room temperature with polyclonal anti-myelin basic protein Ab (Master Diagnostica, Granada, Spain). The immunohistochemical study was done on an Autostainer 480 (Thermo Fisher Scientific Inc., Waltham, MA) using the polymer-peroxidase-based method and developed with diaminobenzidine.

### 2.8. Assessment of Autoreactive T Cell Responses in EAE Mice

Spleen and draining lymph node (DLN) cells from EAE mice treated i.p. with PBS (control, *n* = 4) or allogeneic mASC (*n* = 4) at the onset of disease (clinical scores: 0-1) were isolated 7 days after mASC injection and stimulated with MOG_35-55_ (50 *μ*g/mL) or anti-CD3 (1 *μ*g/mL; 145-2C11; BD Pharmingen, San Diego, CA) at 1.5 × 10^6^ cells/mL in flat bottomed 96-well plates for proliferation and at 10^6^ cells/mL in 24-well for cytokine determination. Supernatants were collected after 48 hours and assayed for cytokine levels by ELISA. After 3 days, cell proliferation was determined by [^3^H]-thymidine uptake as described above. For intracellular cytokine staining, spleen and DLN cell suspensions (1 × 10^6^ cells/mL) were stimulated with PMA (50 ng/mL) and ionomycin (0.5 *μ*g/mL; both from Sigma Aldrich) for 6 hours in the presence of 3 *μ*M monensin (eBioscience, San Diego, CA) during the last three hours. Cells were processed for FACS analysis as described below.

### 2.9. Isolation and Characterization of DCs from EAE Mice

To determine the effect of mASCs on DC phenotype and function in vivo, EAE mice were treated i.p. with PBS (control, *n* = 4) or with allogeneic mASC (*n* = 4) after the onset of disease (clinical scores: 1-2), and 7 days later, DLNs were isolated and digested with 1.6 mg/mL collagenase type IV and 0.1% DNAse I (Sigma Aldrich) in RPMI1640 medium without supplements at 37°C for 30 minutes. For intracellular TNF-*α* staining, DLN cells were washed twice with complete RPMI1640 and 2 × 10^6^ cells/mouse were plated in 12-well plates in the presence of 3 *μ*M monensin for 4 h. Cells were gently harvested using cell scrapers, washed using FACS buffer, and processed for intracellular staining as described below. For TNF-*α* ELISA, CD11c^+^ DCs were immunomagnetically purified using CD11c-microbeads (Miltenyi Biotech, Bergisch Gladbach, Germany) from collagenase type IV-digested DLNs and plated at 2.5 × 10^5^ cells/mL in the presence of LPS (1 *μ*g/mL). Supernatants were collected after 48 hours for cytokine determinations.

### 2.10. Generation of Bone Marrow-Derived DCs (BM-DCs) and mASC Cocultures

BM-DCs were generated as previously described [32]. Briefly, 0.4 × 10^6^ BM cells/mL from C57Bl/6 mice were cultured in complete RPMI1640 containing 20 ng/mL GM-CSF (Peprotech, London, UK). After 6–8 days, nonadherent cells were harvested and CD11c^+^ immature DCs were immunomagnetically purified using CD11c-microbeads (Miltenyi Biotech) according to the manufacturer's instructions. Cell preparations consisted of >95% CD11c^+^ DCs. To assess the effect of mASCs on the maturation of DCs, different numbers of mASCs were plated in 12-well plates and allowed to adhere for at least 6 hours. CD11c^+^ DCs (0.4 × 10^6^ cells/well) were seeded into wells with or without mASCs and LPS (1 *μ*g/mL) was added for 48 hours to induce DC maturation/activation. In some experiments L-NAME (1 mM) or indomethacin (20 *μ*M) was added to the DC: mASC cocultures during maturation. DCs were harvested from the cocultures by gently collecting the nonadherent DCs from the adherent mASC monolayer. The acquired cells consisted of >95% CD11c^+^ DC. Supernatants were collected and assayed for cytokine levels by ELISA or NO_2_ levels by Griess assay. Cell suspensions were processed for flow cytometry analysis of cell surface antigens or used for MLR as described below.

### 2.11. Mixed Leukocyte Reaction

Spleens from BALB/c (H-2^d^) were homogenized and erythrocytes lysed using the Ammonium-Chloride-Potassium (ACK) lysis buffer. Cells (4 × 10^6^ cells/mL) were plated in Petri dishes and allowed to adhere at 37°C for 90 minutes. Nonadherent cells were collected and used as responders. C57Bl/6 (H-2^b^) DCs from the DC: mASC cocultures or purified CD11c^+^ DCs from the DLNs of untreated or mASC-treated EAE mice were added in flat bottomed 96-well plates (15000 DCs/well) together with 3 × 10^5^ responder cells. After 4 days, cell proliferation was determined by ^3^H-thymidine uptake as described above.

### 2.12. Flow Cytometry

Collagenase/DNAse-treated DLN cell suspensions or mASC-cultured DCs were preincubated with anti-Fc*γ*RII/Fc*γ*RIII mAb at 2.5 *μ*g/mL (2.4G2; BD Pharmingen) and 7-aminoactinomycin D (2 *μ*g/mL; Sigma Aldrich) and then stained with the following antibodies: CD11c-APC (N481; eBioscience), CD40-PE (2/23), CD80-PE (16-10A1), and/or CD86-PE (GL-1) (BD Pharmingen). For intracellular cytokine staining cells were fixed, permeabilized, and stained for IFN-*γ*-FITC (XMG1.2), IL-17-PE (TC11-18H10), TNF-*α*-PE (MP6-XT22), IL-4-PE (BVD4-1D11), and IL-10-PE (JES5-16E3) (all from BD Biosciences, San Diego, CA) using the BD cytofix/cytoperm kit (BD Biosciences) according to the manufacturer's instructions. Each sample was stained with appropriate isotype controls. For the analysis of foxp3 expression cells were stained with CD4-FITC (GK1.5; BD Biosciences) and subsequently processed for foxp3 staining using the foxp3 staining kit (eBioscience) according to the manufacturer's instructions. Stained cells were analyzed on a FACSCalibur flow cytometer (BD Biosciences).

### 2.13. Cytokine and PGE_2_ ELISAs

The cytokine content in supernatants from the mASC: splenocyte and DC cultures were determined by sandwich ELISAs using capture/biotinylated detection antibody pairs for IFN-*γ*, TNF-*α*, IL-12p40 (BD Pharmingen), IL-10, IL-17, and CXCL10 (eBioscience). Plates were developed using peroxidase-labelled streptavidin (Sigma) and 2,2′-azino-bis(3-ethylbenzthiazoline-6-sulphonic acid) (ABTS) and read at 405 nm. PGE_2_ levels in supernatants were measured by ELISA (Cayman Chemicals, Ann Arbor, MI) according to the manufacturer's instruction.

### 2.14. Gene Expression Analysis

Total RNA was purified using Ultraspec (Biotecx, Huston, TX) from spinal cords isolated from PBS-treated (control, *n* = 4) or allogeneic mASC-treated (*n* = 4) EAE mice (7 days after treatment) or from mASCs stimulated with LPS (1 *μ*g/mL, Sigma) or TNF-*α* (10 ng/mL, Peprotech) and IFN-*γ* (10 ng/mL, BD Biosciences) for 6, 12, and 24 hours. Total RNA (1 *μ*g/sample) was reverse transcribed using M-MuLV RT (Roche Diagnostic, Basel, Switzerland) and random hexamer primers. Semiquantitative PCR and qPCR were performed using Taq polymerase (Biotools, Madrid, Spain) or Supermix (Bio-Rad, Hercules, CA), respectively. Primer pairs include the following: iNOS FW: 5′-GTTCTCAGCCCAACAATACAAGA-3′; iNOS RV: 5′-GTGGACGGGTCGATGTCAC-3′; MCP-1 FW: 5′-TTAAAAACCTGGATCGGAACCAA-3′; MCP-1 RV: 5′-GCATTAGCTTCAGATTTACGGGT-3′; TGF-*β*1 FW: 5′-TGCGCTTGCAGAGATTAAAA-3′; TGF-*β*1 RV: 5′-AGCCCTGTATTCCGTCTCCT-3′; IL-10 FW: 5′-GGTTGCCAAGCCTTATCGGA-3′; IL-10 RV: ACCTGCTCCACTGCCTTGCT; IDO FW: 5′-GGCTAGAAATCTGCCTGTGC-3′; IDO RV: 5′-AGAGCTCGCAGTAGGGAACA-3′; COX-2 FW: 5′-GGGTTGCTGGGGGAAGAAATGTG-3′, COX-2 RV: 5′-GGTGGCTGTTTTGGTAGGCTGTG-3′; Arginase I FW: 5′-CAGAAGAATGGAAGAGTCAG-3′; Arginase I RV: 5′-CAGATATGCAGGGAGTCACC-3′; Foxp3 FW: 5′-TTCATGCATCAGCTCTCCAC-3′; Foxp3 RV: 5′-CTGGACACCCATTCCAGACT-3′; IFN-*γ* FW: 5′-ACACTGCATCTTGGTTTGC-3′; IFN-*γ* RV: 5′-TTGCTGATGGCCTGATTGTC-3′; *β*-actin FW: 5′-AATCGTGCGTGACATCAAAG-3′; *β*-actin RV: 5′-ATGCCACAGGATTCCATACC-3′.

### 2.15. Statistical Analysis

All results are expressed as mean (SEM) of at least 3 independent experiments unless otherwise stated in the figure legends. The Mann–Whitney *U*-test was applied on all in vivo results and cell-culture experiments to compare nonparametric data for statistical significance. A *p* value < 0.05 was considered significant.

## 3. Results

### 3.1. Immunomodulatory Mechanisms of mASCs In Vitro

**Figure 1:**
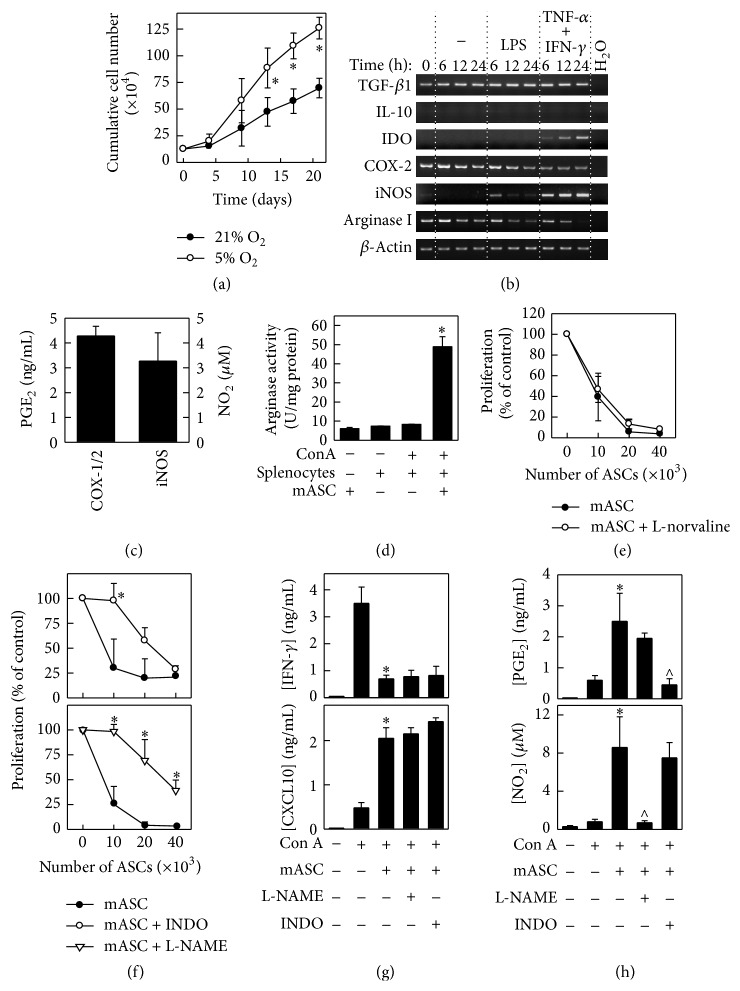
mASCs expanded in hypoxia maintain their immunosuppressive activities. (a) Proliferative response of mASCs expanded in normoxia (21% O_2_) or in hypoxia (5% O_2_). mASCs were seeded at 5000 cells/cm^2^ in T25 flasks and counted at different time points when reaching 70–80% of confluence. Data are shown as mean (SD) of one representative experiment out of three. ^*∗*^*p* < 0.05 versus 21% O_2_. ((b) and (c)) Immune phenotypic characterization of mASCs. mASCs were expanded under hypoxic conditions and then cultured with medium (unstimulated) or stimulated with LPS (1 *μ*g/mL) or TNF-*α* (10 ng/mL) and IFN-*γ* (10 ng/mL) for different time points (24 hours for NO_2_ determination). Gene expression of different immune markers was analyzed using semiquantitative PCR. iNOS activity was measured in TNF-*α*/IFN-*γ*-stimulated mASCs by the detection of NO_2_ in culture supernatants using Griess assay. COX-1/2 activity was determined by measuring the PGE_2_ levels in culture supernatants from unstimulated mASCs. (d) Arginase activity is induced in mASC: splenocyte cocultures. Splenocytes were cultured with or without mASCs and stimulated with ConA for 3 days. Defined quantities of cell lysates were then subjected to an in vitro arginase activity assay. Data are shown as mean (SEM) of 3 independent experiments. ^*∗*^*p* < 0.05 versus splenocytes + ConA. ((e), (f), and (g)) mASCs suppress T cell proliferation in vitro via iNOS and COX-1/2 but not arginase activity. Splenocytes (10^6^ cells/mL) were stimulated with ConA (2.5 *μ*g/mL) in the presence of different numbers of mitomycin C-treated mASCs (ratio 20 : 1 for IFN-*γ* and CXCL10 determination). ((e) and (f)) The iNOS inhibitor L-NAME (1 mM), the COX-1/2 inhibitor indomethacin (20 *μ*M), and the arginase inhibitor L-norvaline (10 mM) were added to cultures when indicated. After 3 days, cell proliferation was measured by [^3^H]-thymidine incorporation and (g) IFN-*γ* and CXCL10 contents in the supernatants were determined by ELISA. Data are shown as mean (SEM) of 3 independent experiments. ^*∗*^*p* < 0.05 versus mASC in (f) and ^*∗*^*p* < 0.05 versus ConA in (g). (h) iNOS and COX-2 activities are increased in mASC: splenocyte cocultures. ConA-stimulated splenocytes (10^6^ cells/mL) were cultured with mitomycin C-treated mASCs (ratio 20 : 1) in the presence of L-NAME or indomethacin for 3 days and the PGE_2_ and NO_2_ contents measured in the culture supernatants. Results are shown as mean (SEM) of 3 separate experiments. ^*∗*^*p* < 0.05 versus ConA; ^∧^*p* < 0.05 versus mASC + ConA.

Acquiring high numbers of low passage MSCs with potent immunosuppressive capacity is crucial for their successful use as a therapy for inflammatory/autoimmune diseases [33]. In agreement with previous studies [34, 35], we found that mASC expanded at low oxygen tension (5% O_2_) proliferated at a higher rate compared to mASCs expanded at normoxia (Figure 1(a)). Thus, we decided to culture the ASCs at 5% O_2_ and use these cells for the subsequent characterization of their immunomodulatory functions in vitro and in vivo. Whereas mASCs constitutively expressed TGF-*β*1, COX-2, and arginase I mRNAs and PGE_2_, the expression of both IDO and iNOS mRNA and NO_2_ production was induced upon stimulation with TNF-*α* and IFN-*γ*, but not LPS (Figures 1(b) and 1(c), Figure S2). Interestingly, we found a significant induction of arginase activity in the splenocyte: mASCs cocultures in comparison to mASCs or splenocytes cultured alone (Figure 1(d)). Although arginase activity has been shown to inhibit the proliferation of T cells [36], we found that the addition of the arginase inhibitor L-norvaline did not significantly revert the suppressive activity of mASCs in concanavalin A- (ConA-) induced splenocyte proliferation (Figure 1(e)). In contrast, inhibition of iNOS activity with L-NAME or of COX-1/2 activities with indomethacin significantly alleviated the mASC-mediated suppression of T cell proliferation (Figure 1(f)). However, none of these inhibitors affected the mASC-mediated suppression of IFN-*γ* or the induction of CXCL10 in these cocultures (Figure 1(g)). Since inhibition of iNOS may decrease COX-2 expression and PGE_2_ secretion [37, 38], we measured the PGE_2_ levels in mASC: splenocyte cocultures treated with L-NAME. We found that L-NAME did not affect the production of PGE2 by mASCs and indomethacin did not modulate their iNOS activity. In summary, these data show that low oxygen tension increases the expansion of mASCs in vitro and that these cells are effective in suppressing immune responses in vitro, using both constitutive (COX-1/2) and inducible (iNOS) mechanisms.

### 3.2. Therapeutic Effect of Allogeneic and Xenogeneic ASC on Chronic EAE

**Figure 2:**
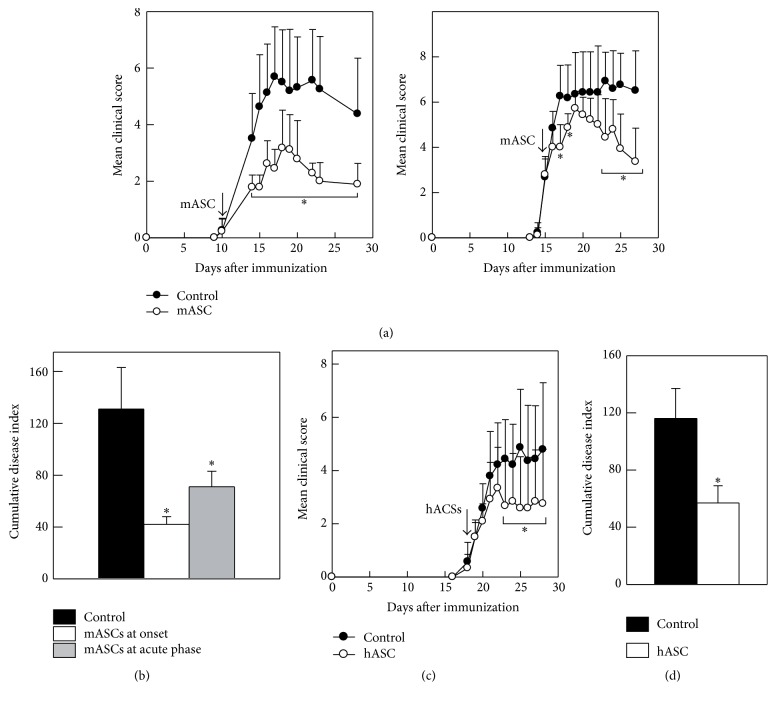
Allogeneic and xenogeneic ASC reduce EAE severity. Chronic progressive EAE was induced in C57Bl/6 mice by immunization with MOG_35-55_. Animals were injected intraperitoneally with PBS (control) or with hypoxia-expanded allogeneic mASCs (10^6^ cells/mouse) either at the onset of EAE (day 11 after immunization) when the mean clinical score was 0.25 in the control group (*n* = 8 mice) and 0.22 in the mASC-treated group (*n* = 9 mice) or at the acute phase of EAE (day 15 after immunization) when the mean clinical score was 2.7 in the control group (*n* = 6 mice) and 2.8 in the mASC-treated group (*n* = 7 mice). (a) Clinical symptoms were scored daily in the group treated at onset (left panel) and at the acute phase of EAE (right panel). (b) Cumulative disease index is the sum of daily clinical scores observed between days 20 and 40. Results are shown as mean (SD). ^*∗*^*p* < 0.05 versus control. (c) Human ASCs ameliorate EAE. Mice with MOG-induced EAE were intraperitoneally injected with PBS (control, *n* = 7) or with hASCs expanded in hypoxia (10^6^ cells/mouse, *n* = 6 mice) during the acute phase of the disease (arrow) when the mean clinical score was 1.5 in both groups. Clinical symptoms were scored daily. (d) Cumulative disease index is the sum of daily clinical scores observed between days 20 and 40. Results are shown as mean (SD). ^*∗*^*p* < 0.05 versus control.

We next wanted to assess the capacity of allogeneic and xenogeneic ASCs to inhibit immune responses in vivo. To this end, we induced chronic EAE in C57Bl/6 mice and administered mASCs to the animals at different time points after immunization. A single injection of allogeneic mASC at the onset or at the acute phase of disease significantly reduced the disease severity compared to control animals receiving PBS (Figure 2(a)). Both early and late injections of mASCs significantly reduced the cumulative disease index (Figure 2(b)). Xenogeneic hASCs injected into EAE mice during the acute phase of the disease (animals with a mean score of 1.5) also reduced the mean clinical score and significantly decreased the cumulative disease index compared to control mice (Figures 2(c) and 2(d)).

### 3.3. Allogeneic mASCs Reduce Demyelinization and Inflammatory Infiltration in the Central Nervous System during EAE Progression

**Figure 3:**
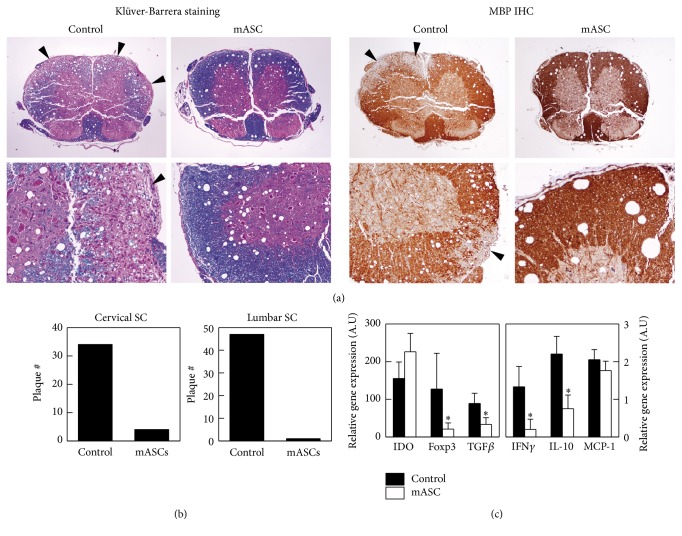
Allogeneic mASCs reduce demyelinization and inflammatory infiltration in the central nervous system during EAE progression. Mice with MOG-induced EAE were intraperitoneally injected with PBS (control, *n* = 4) or with mASCs expanded in hypoxia (10^6^ cells/mouse, *n* = 4) after the onset of the clinical symptoms, and spinal cords were obtained at the peak of the disease 7 days later. ((a) and (b)) Transverse sections of cervical and lumbar regions of spinal cord were randomly analyzed for the presence of inflammatory infiltrates and plaques of demyelinization using the Klüver-Barrera staining (left panels) and anti-myelin basic protein immunohistochemistry (right panels). Arrows point to areas of demyelinization. (c) Gene expression of different inflammatory markers was determined by PCR in mRNAs isolated from the spinal cords. Results are shown as mean (SD) of 4 mice per group. ^*∗*^*p* < 0.05 versus control.

We then wanted to analyze CNS tissue sections from control and mASC-treated EAE mice for the presence of inflammatory infiltrates and demyelinating plaques. To this end, mASCs were injected intraperitoneally into EAE mice after the onset of the clinical symptoms (average score 1.9 (0.4)) and spinal cords were obtained 7 days later from mASC-treated mice (average score 2.2 (1.9)) and control mice (average score 3.5 (1.1)) and processed for staining. We found that mASC-treated mice exhibited little cell infiltration into the CNS parenchyma and subsequently few demyelinating plaques compared to control EAE mice (Figures 3(a) and 3(b)). Consistent with the reduced leukocyte infiltration, we detected lower levels of IFN-*γ*, but also TGF-*β*1, IL-10, and foxp3 mRNAs in mASC-treated mice compared to control EAE mice (Figure 3(c)).

### 3.4. Allogeneic mASCs Inhibit MOG-Specific Immune Responses but Do Not Induce foxp3+ Tregs

**Figure 4:**
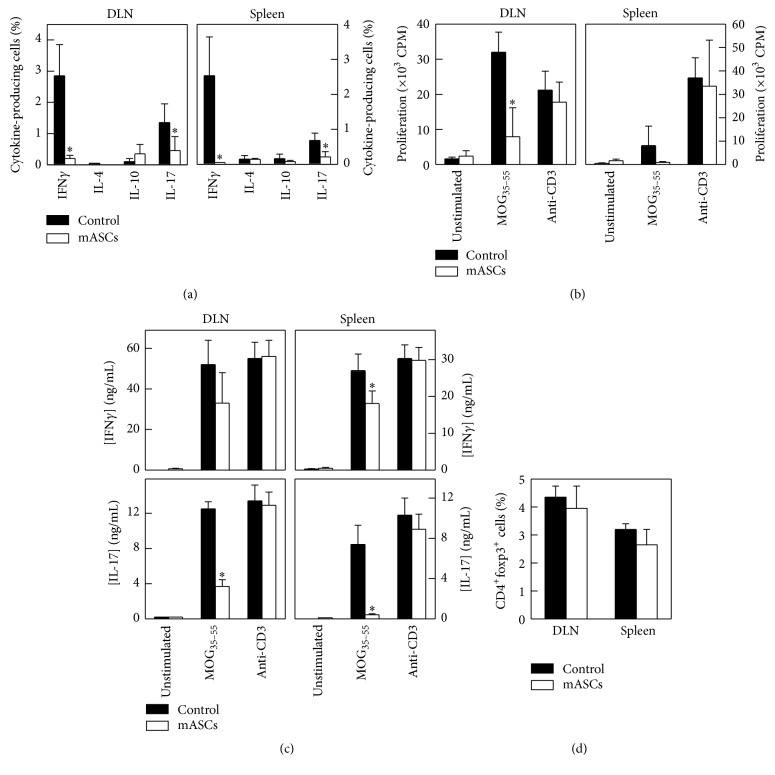
Allogeneic mASCs inhibit MOG-specific immune responses but do not induce foxp3^+^ Tregs. mASCs were injected intraperitoneally in MOG-induced EAE mice after the onset of the clinical symptoms and spleens and DLNs were isolated at the peak of the disease. Untreated EAE mice were used as controls. (a) The percentages of cytokine-producing cells in spleen and DLNs were determined by intracellular flow cytometry as described in Materials and Methods. ((b) and (c)) DLN and spleen cells were cultured with medium (unstimulated) or stimulated with MOG_35-55 _(50 *μ*g/mL) or anti-CD3 (1 *μ*g/mL, used as unspecific stimulation). Proliferation was measured after 3 days by [^3^H]-thymidine incorporation (b). The content of cytokines was determined in culture supernatants after 48 hours (c). (d) The percentages of CD4^+^Foxp3^+^ T cells in spleen and DLNs were determined by flow cytometry. Results are shown as mean (SD) from 4 mice per group. ^*∗*^*p* < 0.05 versus control.

In both EAE and MS, autoreactive Th1 and Th17 cells producing IFN-*γ* and IL-17, respectively, infiltrate the CNS and promote disease, whereas treatments that induce a skewing towards an IL-4 dominated Th2 response generally suppress EAE [39]. mASC treatment significantly reduced the number of IFN-*γ*- and IL-17-secreting cells in both DLNs and spleen in EAE mice (Figure 4(a) and representative gating strategy in Figure S3). Moreover, there was a significant reduction in the MOG_35-55_-specific recall response, with respect to both proliferation and production of IFN-*γ* and IL-17 compared to control EAE mice. The effect was antigen specific since activation of total T cells with anti-CD3 Abs which resulted in similar proliferation and cytokine production by both groups (Figures 4(b) and 4(c)). However, we could not detect an increase in the anti-inflammatory cytokines IL-10 and IL-4 in mASC-treated mice compared to control mice (Figure 4(a)), suggesting that the mASCs did not induce a skewing towards a Th2 response or IL-10 producing Tregs. In addition, we could not detect any change in the size of the CD4^+^foxp3^+^ T cell population in DLNs or spleens of mASC-treated EAE mice compared to control mice (Figure 4(d)).

### 3.5. mASCs Inhibit the Maturation/Activation of Dendritic Cells In Vitro and In Vivo

**Figure 5:**
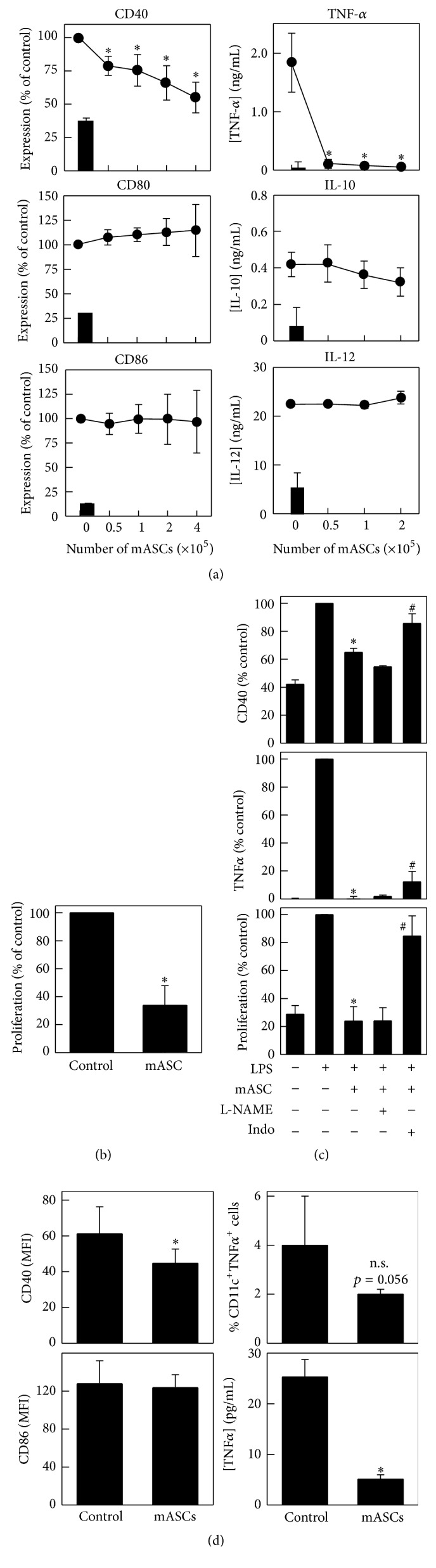
mASCs inhibit the maturation/activation of dendritic cells in vitro and in vivo. (a) mASCs inhibit the maturation of DCs in vitro. Bone marrow-derived DCs from C57BL/6 mice (4 × 10^5^ cells/well) were matured/activated with LPS (1 *μ*g/mL) in the absence or presence of different numbers of mASCs. After 48 hours, nonadherent or loosely adherent cells were harvested (>95% CD11c^+^ DCs) and analyzed for surface expression of CD40, CD80, and CD86, and the culture supernatants were analyzed for TNF-*α*, IL-10, and IL-12 content by ELISA. Data for nonstimulated immature DCs are shown as black bars. Results are shown as mean (SEM) of 3 (CD40, CD80, and CD86 FACS and IL-12 ELISA) or 4 (IL-10 and TNF-*α* ELISA) independent experiments. ^*∗*^*p* < 0.05 versus control (no mASCs). (b) mASC-treated DCs show impaired costimulatory activity on T cells. C57Bl/6 DCs (4 × 10^5^ cells) were LPS-matured in the absence (control) or presence of mASCs (2 × 10^5^ cells) for 48 hours and then added to allogeneic BALB/c splenocytes (used as responders). The proliferation in the MLR was measured by [^3^H]-thymidine incorporation. Results are shown as mean (SEM) of 3 independent experiments. ^*∗*^*p* < 0.05 versus control. (c) COX-1/2 but not iNOS activity is partly responsible for the effect of mASCs on DC maturation. C57Bl/6 DCs (4 × 10^5^ cells) were LPS-matured in the absence or presence of mASCs (2 × 10^5^ cells) and L-NAME (1 mM) or indomethacin (20 *μ*M) for 48 hours. Expression of CD40 was determined in CD11c^+^ cells by flow cytometry (5 independent experiments) and the levels of TNF-*α* in culture supernatants were measured by ELISA (3 independent experiments). The costimulatory activity of the mASC-treated DCs was determined in a MLR using BALB/c splenocytes as responders (4 independent experiments). Results are expressed as percentage of values found with control samples treated with LPS alone in the absence of mASCs and shown as mean (SEM) of the independent experiments. ^*∗*^*p* < 0.05 versus control; ^#^*p* < 0.05 versus mASCs. (d) mASC treatment impairs DC function in DLNs of EAE mice. C57BL/6 mice with initial EAE symptoms (scores 1-2) were injected intraperitoneally with allogeneic mASCs (10^6^ cells/mouse) isolated from BALB/c mice. After 7 days, DLN cells from untreated (mean score 4.6 (1.0)) and mASC-treated mice (mean score 2.1 (1.0)) were analyzed for the expression of surface CD11c, CD40 (*n* = 8 mice/group), and CD86 (*n* = 5 mice/group) and intracellular TNF-*α* (*n* = 4 mice/group) by flow cytometry as described in Materials and Methods. CD11c^+^ DCs were purified by magnetic separation from DLNs from untreated (control) or mASC-treated mice (*n* = 4 mice/group), restimulated (2.5 × 10^5^ cells/mL) with LPS (1 *μ*g/mL) for 24 hours, and the TNF-*α* levels in supernatants are determined by ELISA (lower right panel). Results are shown as mean (SD). ^*∗*^*p* < 0.05.

Considering the importance of DCs in the initiation of the autoimmune response seen in EAE and MS [40], we decided to evaluate the effect of mASC on the DC activation in vitro and in vivo. To this end, we cocultured BM-derived immature DCs with different numbers of mASCs in the presence of LPS. We found that mASC significantly inhibited the LPS-induced expression of CD40 and TNF-*α* while not affecting the induction of CD80 and CD86 or the production of IL-10 or IL-12 by BM-DCs (Figure 5(a) and representative gating strategy in Figure S4). This partial block of DC maturation resulted in a significant decrease in their T cell activating capacity (Figure 5(b)). Since both iNOS and PGE_2_ inhibited the proliferation of splenocytes in vitro and can modulate DC activation [41–44], we set out to investigate their involvement in the mASC-mediated inhibition of DC maturation. We found that addition of indomethacin but not L-NAME significantly reversed the mASC-mediated suppression of TNF-*α* production and CD40 expression resulting in a significant increase in their immunostimulatory capacity (Figure 5(c)). These effects were observed despite the presence of iNOS activity in the mASC: DC cocultures (Figure S2).

To study DC activation in vivo, we purified DCs from DLNs of mASC-treated and control EAE mice 7 days after mASC injection and analyzed their expression of costimulatory markers and TNF-*α* production. Consistent with the in vitro data, DCs from mASC-treated EAE mice expressed significantly lower levels of CD40, but not CD86, compared to DCs from control EAE mice (Figure 5(d)). In addition, intracellular staining of TNF-*α* on whole LN suspensions revealed that mASC-treated EAE mice had fewer CD11c^+^TNF-*α*^+^ DCs compared to control EAE mice (Figure 5(d) and representative dot plots in Figure S5). In order to further analyze DC function we purified CD11c^+^ cells from the DLNs of control and mASC-treated EAE mice and assessed their TNF-*α* producing capacity upon LPS restimulation in vitro. We found that DCs from mASC-treated animals produced less TNF-*α* upon LPS stimulation in vitro compared to control DCs (Figure 5(d)). Taken together, these data suggest that mASC could suppress EAE partly by preventing DC function in vivo.

## 4. Discussion

MSCs have emerged as a promising therapy for MS with some studies suggesting an MSC-mediated modulation of the aberrant immune response [17, 21]. However, the efficacy has so far been modest warranting the search for improved MSC culture protocols and a greater understanding of the mechanisms behind the immunomodulatory and trophic effects of MSCs in vitro and in vivo [16, 17, 19]. Our analysis of the immunomodulatory mechanisms employed by mASCs cultures at 5% O_2_ showed that both their iNOS and COX-1/2 activities are involved in the inhibition of T cell proliferation in vitro. Although iNOS is used only by murine MSCs, COX-1/2 activity has been shown to be important for the immunomodulatory capacity of both murine and human MSCs [29, 45, 46]. In addition to iNOS and COX-1/2, we also detected a significant induction of arginase activity in the mASC: splenocyte cultures, which was found predominantly in the adherent splenocyte fraction and not in the mASC population (Figure S6). This is in agreement with our recent finding that MSCs can induce arginase I^+^ regulatory macrophages in vitro [29]. Although arginase activity does not appear to be important for the MSC-mediated inhibition of T cell proliferation in vitro, the role of MSC-educated macrophages and/or microglia in controlling autoimmune-mediated inflammation in the CNS remains to be elucidated [47, 48].

In agreement with previous studies [11, 27], we found that early injection of mASCs reduced the activation of MOG_35-55_-specific T cells and significantly inhibited EAE progression and severity.

Regulatory T cells (Tregs) control the severity of EAE through the prevention of T cell expansion in the DLNs [49] and by inhibiting activated T cells in the CNS [50]. Several studies have reported an increased frequency of Tregs in the LNs of EAE animals treated with MSCs [26, 51]. Interestingly, two studies observed an increase in Treg number [17] or foxp3 mRNA [21] in PBMCs from MS patients after MSC administration. In contrast, we did not observe an increased frequency of CD4^+^foxp3^+^ T cells or IL-10^+^ cells in spleen and lymph nodes nor increased foxp3 mRNA levels in the CNS after mASC injection into EAE mice which is in accordance with other studies on MSCs and EAE [27, 52, 53]. These discrepancies could be due to differences in the immunomodulatory mechanisms employed by murine and human MSCs, underlying differences in the pathophysiological mechanisms between EAE and MS, and the timing of MSC injection relative to disease progression and to the numbers of MSCs reaching the DLNs [26, 51]. These differences could also partially explain why the clinical efficacy of MSCs in MS is not quite established. Importantly, in another MS-trial, Connick et al. [54] observed a cessation of the protective effects six months after intravenous infusion of MSC, suggesting that a possible induction of Tregs by MSCs was not enough to restore immune homeostasis in MS patients. Future studies are needed to elucidate if/how MSCs can induce functional Tregs in human MS patients and to design strategies to increase their induction.

DCs are specialized antigen presenting cells that, upon maturation by toll like receptor ligands and cytokines, upregulate costimulatory molecules (CD40, CD80, and CD86) and produce cytokines (TNF-*α*, IL-12), in order to induce appropriate T cell responses [55]. However, DCs also play fundamental roles in the induction of EAE and the severity of CNS damage correlates with the activation status of DCs [56–58]. In MS patients, DCs can be found in CNS lesions [59] and circulating DCs exhibit an activated CD40^high^TNF-*α*^+^ phenotype [60]. Importantly, several first-line drugs for MS, such as IFN-*β* [61] and glatiramer acetate [62], inhibit DC function, suggesting that DCs could represent a target for therapeutic modulation. It is thus important to understand if and how MSCs can affect DC function both in vitro and in vivo. PGE_2_ has been shown to inhibit T cell proliferation directly or indirectly through its effect on DCs [63]. Spaggiari et al. [43] showed that BM-MSC-derived PGE_2_ could inhibit early DC differentiation but not LPS-induced DC maturation. In contrast, Yañez et al. [64] described that hASC inhibited DC maturation in a PGE_2_-independent manner. We found that the mASC-derived COX-1/2, but not iNOS activity, could inhibit the LPS-induced CD40 and TNF-*α* expression by DCs. These contradictory results could be due to species differences, source of DCs, and methodological setup as well as differences in the readout for the DC maturation state. We found that although the expression of CD80, CD86, IL-12, and IL-10 was unaffected, mASC-educated DCs were significantly less effective in inducing T cell proliferation. This is in accordance with the study reported by Sá-Nunes et al. [65] showing that exogenous PGE_2_ could inhibit the LPS-induced TNF-*α* secretion and CD40, but not CD80 and CD86, expression on DCs which resulted in a decreased T cell-stimulatory ability. In addition to PGE_2_, other COX1/2-dependent prostanoids, including PGD_2_ and its metabolite 15-deoxy-delta12,14-PGJ_2_ have been shown to inhibit LPS-induced production of proinflammatory cytokines by DCs [66, 67]. Although it is conceivable that PGE_2_ is the main suppressive prostanoid produced by MSCs, further studies should assess the presence of other prostanoids and PGE_2_-derived metabolites [68] and include specific inhibitors for the PGE_2_ receptors, EP1-4. Interestingly, a recent study showed that BM-MSCs can produce PGE_2_ and prostacyclin (PGI2) [69]. The expression of both anti-inflammatory prostanoids was lost upon oncogenic transformation of the BM-MSCs and this was paralleled with their inability to inhibit immune responses in vitro and in vivo. However, the role of BM-MSC-derived PGI2 in the modulation of DC activation has not been investigated.

As discussed above, MSCs can inhibit DC function in vitro but whether this is also true in vivo is less known. To date only one clinical trial has assessed the effects of MSCs on DCs in vivo and found a reduction in circulating CD40+ myeloid DCs shortly after MSC injection [17]. Using a mouse model based on the adoptive transfer of T cells and LPS-activated DCs, Chiesa et al. showed that BM-MSCs could inhibit the migration and T cell priming capacity by the injected DCs in vivo [70]. Another study using a model of bacteria-induced hepatitis showed that MSC administration induced a population of CD80^low^CD86^low^ tolerogenic DCs in vivo [71]. However, only two studies have analyzed the activation state of endogenous DCs in CFA/MOG-induced EAE after MSC infusion with contradictory results [27, 53]. In our study, we found that CD11c^+^ DCs from DLNs of mASC-treated mice expressed lower levels of CD40, but not CD86, and intracellular TNF-*α* compared to EAE control mice corroborating our in vitro data. In addition, purified LN-DCs from mASC-treated mice produced less TNF-*α* upon LPS restimulation in vitro. These data suggest that MSCs can inhibit the activation of DCs in vivo. Although not addressed in the current study, we and others have found that injected MSCs reach DLNs in CFA/MOG-induced EAE [27, 52]. Thus, we believe that the reduction of activated DCs in the DLNs of MSC-treated mice is most likely a combination of (i) reduced DC migration from the immunization site and (ii) a direct MSC-mediated inhibition of DC activation in the DLNs, although the relative contribution of both mechanisms requires further studies.

## 5. Conclusions

Our data suggest that allogeneic ASCs expanded in hypoxia could represent a promising treatment of autoimmune diseases, including MS, through their effects on DC activation and autoimmune responses. It is becoming clear that DCs represent important effector cells, mediating the beneficial effects of MSCs in vivo. Further studies should address in more detail the effects of MSCs on DC function in MS patients and how to increase the MSC-mediated inhibition of DCs. Moreover, the elucidation of the molecular mechanisms involved in the immunomodulatory activity of ASCs, especially the activation of COX-2, suggests that the administration of COX-2 inhibitors (i.e., indomethacin or ibuprofen) during the treatment with ASCs of patients with MS or other autoimmune disorders could impair their therapeutic efficacy.

## Supplementary Material

S1 Fig. Mitomycin-C treatment does not affect to suppressive activity of mASCs.
S2 Fig. mASCs and DCs express iNOS upon stimulation.
S3 Fig. Representative dot plots showing the intracellular staining of IFN-γ and IL-17 in DLN cells from EAE and mASC-treated mice.
S4 Fig. Representative dot plots showing the expression of CD40, CD80 and CD86 on LPS-stimulated CD11c^+^ DCs cultured with or without mASCs.
S5 Fig. Representative dot plots showing intracellular TNF-α in DCs isolated from EAE or mASC-treated mice.
S6 Fig. Arginase activity is induced in mASC:splenocyte cocultures.
