# Supplementary material for: Allogeneic Adipose-Derived Mesenchymal Stromal Cells Ameliorate Experimental Autoimmune Encephalomyelitis by Regulating Self-Reactive T Cell Responses and Dendritic Cell Function
Source: Stem Cells Int. 2017 Jan 30;2017:2389753. doi: 10.1155/2017/2389753 (PMC5303870; doi:10.1155/2017/2389753)
Supplement: Supplementary file 1 — S1 Fig. Mitomycin-C treatment does not affect to suppressive activity of mASCs. S2 Fig. mASCs and DCs express iNOS upon stimulation. S3 Fig. Representative dot plots showing the intracellular staining of IFN-γ and IL-17 in DLN cells from EAE and mASC-treated mice. S4 Fig. Representative dot plots showing the expression of CD40, CD80 and CD86 on LPS-stimulated CD11c+ DCs cultured with or without mASCs. S5 Fig. Representative dot plots showing intracellular TNF-α in DCs isolated from EAE or mASC-treated mice. S6 Fig. Arginase activity is induced in mASC:splenocyte cocultures. [file 2389753.f1.pdf]

Supplementary Figure 1 (S1 Fig)

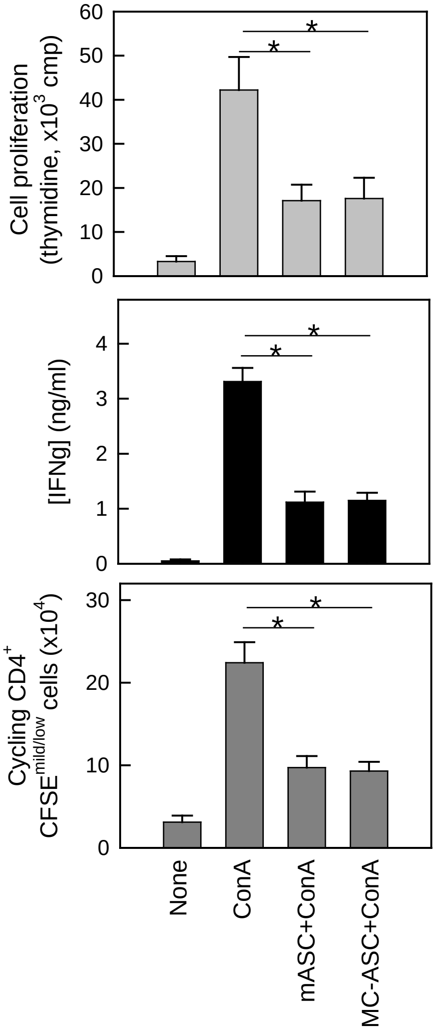

Supplementary Figure 2 (S2 Fig)

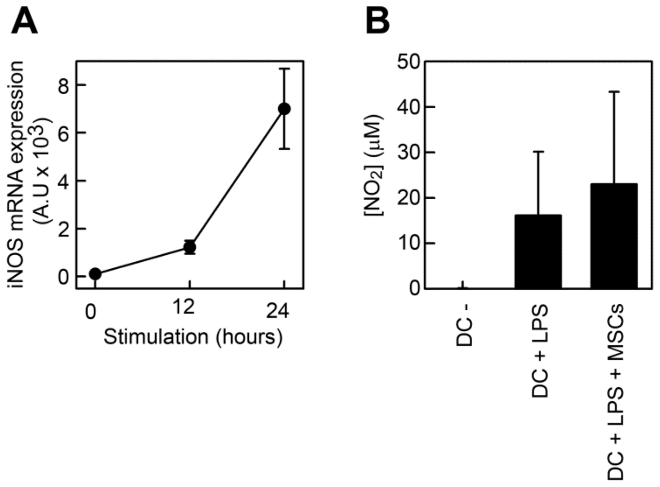

Supplementary Figure 3 (S3 Fig)

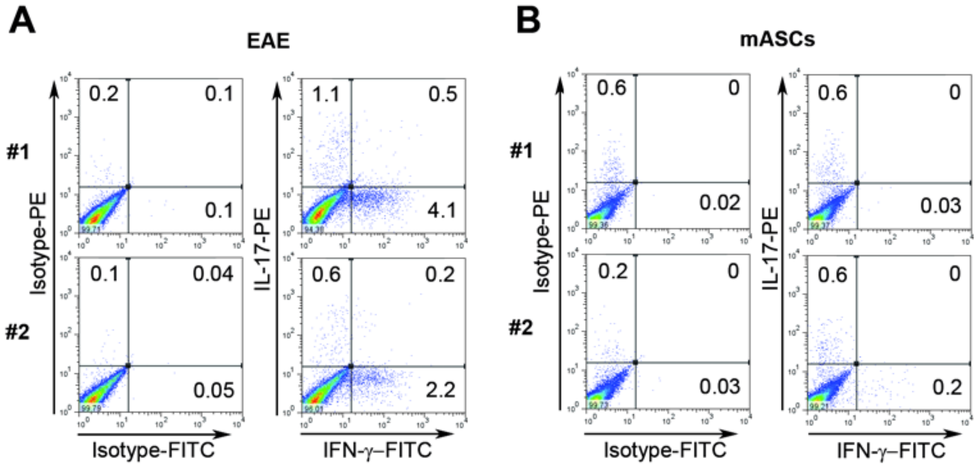

Supplementary Figure 4 (S4 Fig)

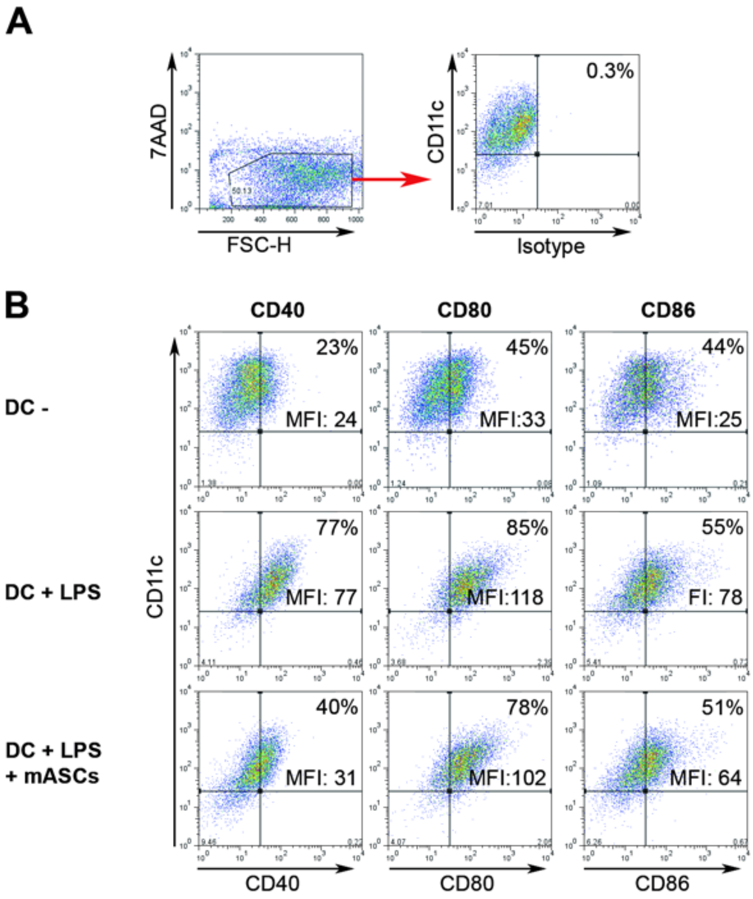

Supplementary Figure 5 (S5 Fig)

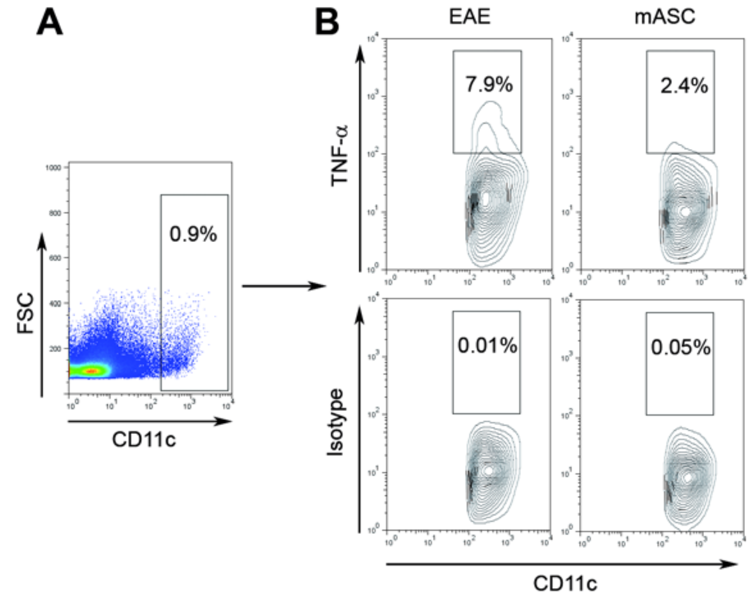

Supplementary Figure 6 (S6 Fig)

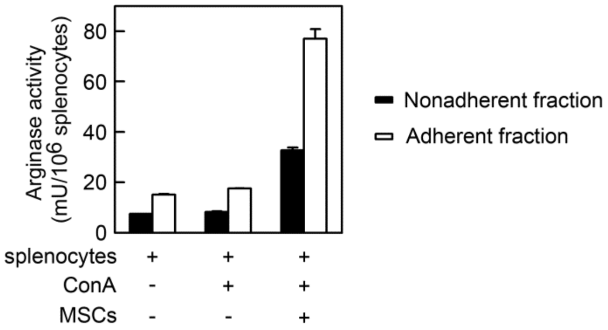

Supporting information - Figure legends

S1 Fig. Mitomycin-C treatment does not affect to suppressive activity of mASCs.

Upper panel: Balb/c splenocytes (2 x 10^5) were added to plates with or without mASCs (10^4) that were pretreated (MC-mASC) or not (mASC) with mitomycin C (50 µg/ml). ConA (2.5 µg/ml) was added to cultures as a mitogenic stimulus of T cells. After 3 days, cell proliferation was determined by [^{3}H]-thymidine incorporation. *Middle panel:* After 3 days, IFN-γ content in the supernatants was determined by ELISA. *Lower panel:* In similar experiments, splenocytes were labelled with 2.5 µM carboxyfluorescein diacetate succinimidyl ester (CFSE, Molecular Probes) prior to setting up cocultures. After culture, cells were labelled with PerCP-conjugated anti-CD4 and fixed with 1% paraformaldehyde, and proliferating cells were determined by CFSE dilution in the CD4+ population by flow cytometry. The number of cycling cells was calculated as the percent of CFSE$^{mild/low}$ cells that had divided by the total number of cells. Data are shown as mean (SEM) of 3 independent experiments. * p<0.05 between groups.

S2 Fig. mASCs and DCs express iNOS upon stimulation

iNOS is induced in mASCs upon cytokine stimulation and in mASC:DC cocultures. (A) mASCs were stimulated with IFN-γ (10 ng/ml) and TNF-α (10 ng/ml) for 12 and 24 hours. The level of iNOS mRNA at each time point was measured using qRT-PCR. Data is shown as mean(SEM) of two independent experiments. (B) Bone-marrow derived DCs (1 x 10^6) were activated with LPS (1 µg/ml) in the absence or presence of mASCs (0.5 x 10^6) for 48 hours and the levels of NO_2 in the culture supernatant were

measured using Griess assay as described in the materials and methods. Data is shown as mean (SEM) of two independent experiments.

S3 Fig. Representative dot plots showing the intracellular staining of IFN-γ and IL-17 in DLN cells from EAE and mASC-treated mice.

PMA/Ionomycin-stimulated lymph node cells from EAE (A) and mASC-treated (B) mice were fixed and stained intracellularly with the appropriate isotype controls (A and B, left dot plots) and antibodies against IL-17 and IFN-γ (A and B, right dot plots) and analyzed on a FACSCalibur flow cytometer. The figure shows data from two individual mice (#1 and #2) from each group. The percentage of IFN-γ^+ and IL-17^+ cells were calculated as % IFN-γ-FITC$^+$ cells - % Isotype-FITC$^+$ cells and % IL-17-PE$^+$ cells - % Isotype-PE$^+$ cells, respectively.

S4 Fig. Representative dot plots showing the expression of CD40, CD80 and CD86 on LPS-stimulated CD11c$^+$ DCs cultured with or without mASCs.

CD11c$^+$ DCs were cultured alone, with or without LPS, or with mASCs (0.4 x 10^6 cells) and LPS for 48 hours as described in the materials and methods. Non-adherent cells were gently harvested and stained with (A) 7AAD, CD11c-APC and isotype-PE or (B) CD40-PE (left column dot plots) or CD80-PE (center column dot plots) or CD86-PE (right column dot plots). The graph shows representative data from one out of 3 independent experiments. The values represent the percentage of CD40-, CD80- and CD86-positive CD11c$^+$ DCs (relative to the isotype control) and the mean fluorescence intensity (MFI) of CD11c$^+$ DC population stained for CD40, CD80 and CD86.

S5 Fig. Representative dot plots showing intracellular TNF-α in DCs isolated from EAE or mASC-treated mice.

The draining LNs were harvested from EAE and mASC-treated mice and stained for CD11c and intracellular TNF-α as described in the materials and methods. The general gating of $CD11c^+$ DCs is shown in (A) and the gated DCs were analyzed for intracellular TNF-α (B, top panels) and isotype control staining (B, lower panels).

S6 Fig. Arginase activity is induced in mASC:splenocyte cocultures

Splenocytes were left to adhere to plastic for 90 minutes at 37°C. Non-adherent (black bars) and plastic adherent (white bars) splenocytes were then harvested and added to mASCs and stimulated with ConA (1 µg/ml). After 72 hours, cells were lysed and the arginase activity in the lysates was analyzed as described in materials and methods.
